# Analysis of risk factors of bladder neck contracture following transurethral surgery of prostate

**DOI:** 10.1186/s12894-021-00831-6

**Published:** 2021-04-11

**Authors:** Yi-Zhong Chen, Wun-Rong Lin, Yung-Chiong Chow, Wei-Kung Tsai, Marcelo Chen, Allen W. Chiu

**Affiliations:** 1 Department of Urology, MacKay Memorial Hospital, No. 92, Sec. 2, Zhongshan N. Rd., Zhongshan Dist., Taipei, 104 Taiwan; 2 School of Medicine, MacKay Medical College, New Taipei City, Taiwan; 3 Department of Cosmetic Applications and Management, MacKay Junior College of Medicine, Nursing and Management, Taipei, Taiwan; 4 School of Medicine, National Yang-Ming University, Taipei, Taiwan

**Keywords:** Transurethral resection of prostate, Bladder neck contracture, Benign prostate hypertrophy, Thulium enucleation/vaporesection/vapoenucleation of prostate

## Abstract

**Backgrounds:**

The aim of the present study was to investigate the perioperative parameters associated with bladder neck contracture (BNC) after transurethral surgery of the prostate and to compare the incidence of BNC after transurethral resection of the prostate (TURP) or Thulium vaporesection (resection group) versus Thulium vapoenucleation or enucleation of the prostate (enucleation group).

**Methods:**

Between March 2008 and March 2020, 2363 patients received TURP and 1656 patients received transurethral surgery of the prostate with Thulium laser (ThuP) at Mackay Memorial Hospital. A total of 62 patients developed BNC. These BNC patients were age-and operation-matched to 124 randomly sampled TURP/ThuP controls without BNC. A 1:1 propensity score matching model was used to evaluate the difference in incidence of BNC.

**Results:**

Our study demonstrated that a greater proportion of BNC patients had history of cerebrovascular accidents (11/62 vs. 7/124, p = 0.009), coronary artery disease (14/48 vs. 16/108, p = 0.03), chronic kidney disease (14/62 vs. 11/124, p = 0.01), and two or more comorbidities (29/62 vs. 27/124, p = 0.001) compared with NBNC patients. Multivariate analysis showed that smaller prostate volume (OR 0.96 (0.94–0.99), p = 0.008) and recatherization (OR 5.6 (1.02–30.6), p = 0.047) were significantly associated with BNC. A ROC curve predicted that a prostate volume < 42.9 cm^3^ was associated with a notably higher rate of BNC. The propensity score matching model reported there was no difference in incidence between resection and enucleation groups.

**Conclusion:**

This study demonstrated that incidence of BNC was the same in different surgical techniques and that low prostate volume, recatherization and ≥ 2 comorbidities were positively correlated with the development of BNC after TURP or ThuP.

**Supplementary Information:**

The online version contains supplementary material available at 10.1186/s12894-021-00831-6.

## Introduction

Benign prostate hyperplasia (BPH) is a common problem among older males. As life expectancy increases, BPH has become a common problem [1]. BPH with lower urinary tract symptoms has a notable impact on daily life and can lead to severe genitourinary problems. When surgically indicated, bipolar transurethral resection of prostate (TURP) and transurethral surgery of the prostate with Thulium laser (ThuP), which are the major endoscopic surgery options, are typically performed, and they have been shown to have favorable outcomes.

Improvement in surgical instruments and technology have resulted in decreased intra-operative and post-operative complications. Bleeding is the most commonly observed intra-operative complication, while dysuria is a bothersome post-operative complication [2, 3]. Post-operative dysuria can often be caused by urethra stricture or bladder neck contracture (BNC). The incidence of BNC in the medical literature has been reported to range from 2.2 to 9.8% [4].

The underlying mechanism of BNC is not currently well understood [5]. Many risk factors have been associated with BNC, such as untreated preoperative urinary tract infection, small prostate volume, unsuitable resectoscope diameter, long operative time, history of prostatitis and recatherization after surgery [5–7]. The aim of the current study was to investigate the perioperative parameters associated with the occurrence of BNC after TURP or ThuP, and the difference in BNC incidence rates between different techniques, resection and enucleation.

## Methods

We retrospectively reviewed the medical records of patients who underwent either TURP or ThuP at Mackay Memorial Hospital between March 2008 and March 2020. 16 surgeons performed the surgery and they were all experience in transurethral surgery. A total of 2363 patients underwent TURP and 1656 patients ThuP. Among ThuP, 458 patients received Thulium vaporesection (ThuVap) and 1158 patients received Thulium vapoenucleaiton of the prostate (ThuVep) and Thulium enucleation of the prostate (ThuEp). Patients with primary bladder neck stenosis, active infection before surgery, history of previous endoscopic surgery of the urinary tract, or incomplete data were excluded from this study. A total of 66 patients found to have BNC after the procedure and a total of 3953 patients didn't have BNC after the procedure. Four were excluded due to lack of PSA data, and therefore a total of 62 patients were included in the study. BNC occurred after TURP in 35 patients and after ThuP in 27 patients. They all subsequently received bladder neck incision at a later date. BNC was suspected if patients complained of progressive dysuria or gradually decreasing maximal urinary flow (less than 10 mL/s in the urodynamic study). Definite diagnosis was made by cystoscopy. Upon confirmation of the diagnosis of BNC, bladder neck incision was performed using bipolar instruments (energy set at 160 J for cutting and 80 J for coagulation). Due to low incidence rate (1.6%, 66/4019) of BNC, we designed a 1:2 case control cohort. These 62 BNC patients (BNC group, 35 TURP 10 ThuVap and 17 ThuVep or ThuEp) were age-and operation-matched to 124 randomly sampled controls without BNC (NBNC group, 70 TURP, 20 ThuVap and 34 ThuVep or ThuEp). There was no statistically significance in age and operation between BNC and NBNC group (Table 1; Additional file 1: Table S1). Propensity score matching model was used to minimize the bias in patients between resection group (N = 135, 105 TURP and 30 ThuVap) and enucleation group (N = 51, ThuVep or ThuEp). We conducted a 1:1 propensity score matching model and propensity score was set at 0.01.

**Table 1:** Patients’ characteristics between the BNC and NBNC groups

	TURP		P value	ThuP		P value	Total		P value
	BNC (N = 35)	NBNC (N = 70)		BNC (N = 27)	NBNC (N = 54)		BNC (N = 62)	NBNC (N = 124)	
Age (years)	68.95 ± 11.04	68.47 ± 10.80	0.83	71.96 ± 7.92	71.44 ± 7.82	0.78	70.26	69.67	0.746
Prostate volume (ml)	40.46 ± 20.29	55.05 ± 18.40	0 < 0.001*	46.37 ± 16.83	60.03 ± 21.42	< 0.001*	43.0 ± 18.95	57.2 ± 19.84	0 < 0.001*
PSA (ng/ml)	5.17 ± 7.02	9.69 ± 15.5	0.10	11.76 ± 22.92	8.12 ± 11.85	0.35	8.04 ± 16.19	9.0 ± 14.02	0.67
Maximal urinary flow (ml/s)	11.24 ± 10.20	7.89 ± 6.01	0.04*	9.43 ± 7.01	8.79 ± 5.87	0.67	10.4 ± 8.84	8.28 ± 5.94	0.05
Post-voiding residual urine (ml)	154.6 ± 194.94	141.51 ± 177.94	0.73	110.25 ± 147.49	97.71 ± 150.84	0.72	135.29 ± 175.91	122.43 ± 167.45	0.39
Foley insertion before TURP or ThuP (yes/no)	9/26	13/57	0.40	5/22	9/45	0.84	14/48	22/102	0.43
Preoperative Hemoglobin (gm/dl)	14.05 ± 1.14	14.16 ± 1.58	0.73	13.63 ± 0.99	14.10 ± 1.30	0.12	13.87 ± 1.09	14.12 ± 1.45	0.22
Postoperative Hemoglobin (gm/dl)	13.41 ± 1.37	13.54 ± 1.56	0.68	13.00 ± 0.90	13.32 ± 1.29	0.23	13.22 ± 1.20	13.44 ± 1.44	0.65
Hemoglobin change (gm/dl)	0.67 ± 0.56	0.62 ± 0.67	0.70	0.59 ± 0.50	0.76 ± 0.76	0.29	0.64 ± 0.53	0.68 ± 0.71	0.65
Prostate resection weight (g)	11.09 ± 16.0	16.0 ± 10.86	0.02*	12.37 ± 14.94	17.65 ± 15.09	0.14	11.64 ± 11.75	16.67 ± 12.84	0.01*
Percentage of prostate resected (%)	29.2 ± 15.63	27.98 ± 13.96	0.69	24.76 ± 23.42	27.01 ± 16.67	0.62	27.2 ± 19.36	27.5 ± 15.13	0.91
Surgical time (min)	85.11 ± 100.93	100.93 ± 31.69	0.01*	93.63 ± 35.20	111.94 ± 37.11	0.04*	43.0 ± 30.9	57.2 ± 34.5	0.001*
Resection speed (g/min)	0.12 ± 0.06	0.15 ± 0.09	0.04*	–	–	–	–	–	–
Hospital stay (days)	3.14 ± 0.49	3.19 ± 0.57	0.71	3.00 ± 0.28	3.13 ± 0.48	0.20	3.08 ± 0.42	3.16 ± 0.53	0.30
Smoking (yes/no)	17/18	25/45	0.21	7/20	9/45	0.32	24/38	34/90	0.12
Hypertension (yes/no)	18/17	32/38	0.58	17/10	24/30	0.12	35/27	56/68	0.15
Diabetes mellitus (yes/no)	7/28	10/60	0.45	7/20	8/46	0.23	14/48	18/106	0.17
Cerebrovascular accidents (yes/no)	7/28	5/65	0.05	4/23	2/52	0.07	11/51	7/117	0.009*
Coronary artery disease (yes/no)	6/29	10/60	0.70	9/18	6/48	0.02*	14/48	16/108	0.09
Chronic kidney disease (yes/no)	5/30	8/62	0.68	9/18	3/51	0.001*	14/48	11/113	0.01*
Number of comorbidities (≥ 2/ ≤ 1)	14/21	16/54	0.07	15/12	11/43	0.001*	29/33	27/97	0.001*
Prostate cancer (yes/no)	6/69	6/64	0.193	2/25	4/50	1	8/54	10/114	0.29
Other malignancy (yes/no)	5/30	3/67	0.07	1/26	1/53	0.61	6/56	4/54	0.09
Concurrent ESCL (yes/no)	2/33	4/66	1	0/27	4/50	–/–	2/60	8/116	0.36
Recatherization after surgery (yes/no)	4/31	0/70	–/–	3/24	2/52	0.19	7/55	2/122	0.004*
Post-opertion urinary tract infection (yes/no)	1/34	1/69	0.61	2/25	0/54	–/–	3/59	1/123	0.11

* means  p < 0.05

The standard procedures for TURP and ThuP was: 1) urethral sounding from 16 French (Fr) to 32 Fr. 2) Insertion of a 24 Fr resectoscope in a 26 Fr sheath. 3) Resection of the prostate piece by piece using bipolar instruments (energy set at 160 J for cutting and 80 J for coagulation) or vaporesection or enucleation of the prostatic adenoma with a 120 W-Thulium laser (Vela XL or RevoLix 120, energy set at 20–40 W for enucleation and 60–80 W for resection). 4) Resected tissue was then morcellated or evacuated. 5) Final hemostasis was achieved with bipolar instruments (160 J for cutting and 80 J for coagulation). During TURP and ThuP, normal saline was used as the flushing fluid during surgery. A 22 Fr urethral triple-lumen Foley catheter was placed for traction for 8 h post-operation and for continuous irrigation with sterile saline for 24 h post-operation. The Foley was removed in 48–72 h post-operatively. Antibiotic prophylaxis with first generation cephalosporin was administered pre-operatively and 12 h post-operatively. Patients were then prescribed oral first-generation cephalosporin for a week.

We reviewed patient medical records to obtain the preoperative prostate specific antigen (PSA) level, smoking history, complete hemochemical data (including white blood cell count, red blood cell count, platelet count, renal and liver function, and electrolytes), prostate volume (measured by transrectal ultrasound), pathology, surgical time, uroflowmetry, comorbidities (hypertension, diabetes mellitus, coronary artery disease, cerebrovascular accident, chronic kidney disease and other malignancy), concomitant endoscopic cystolithotripsy (ESCL), capsule perforation during surgery, the whether or not a Foley was inserted before surgery, postoperative complication (urinary tract infection according to urine culture and epididymitis/orchitis, prostate bleeding and blood transfusion), postoperative pathology (benign prostate hyperplasia or prostate cancer), recatherization after surgery. The percentage of prostate resection was defined as the prostate specimen weight/prostate volume. Resection speed was defined as prostate specimen/surgical time (g/minutes) in patients receiving TURP. Resection speed could not be evaluated because a significant amount of time was spent in morcellation during ThuP. Chronic kidney disease was defined as an eGFR < 60 ml/min/1.73 m^2^ or creatinine > 1.2 mg/dl.

All data in the case control study between BNC and NBNC were compared using Student’s t-tests, chi-squared tests, univariate and multivariate logistic regression analysis. All data in the propensity score matching model between resection and enucleation groups were compared using paired t test, McNemar’s test and logistic regression analysis. IBM SPSS Statistics 25.0 was used for data analysis. Statistical significance was set at p < 0.05.

Use of the data in the current study was approved by the Institutional Review Board of Mackay Memorial Hospital. All personal information was de-identified prior to data analysis, thus ensuring patient confidentiality.

## Results

Our study showed that 66 patients developed BNC between 2008 and 2020. The incidence of BNC was 1.7% (39/2363) after TURP and 1.6% (27/1656) after ThuP (2.2% after ThuVap (10/458), and 1.4% (17/1198) after ThuVep). The average time to a diagnosis of BNC was 21.3 months (range from 2 to 130 months, IQR: 19 months).

Sixty-two BNC patients with complete data (35 TURP, 10 ThuVap and 17 ThuVep or ThuEp) were randomly age-and operation-matched in a 1:2 ratio with 124 patients without BNC (70 TURP, 20 ThuVap and 34 ThuVep or ThuEp). The average age of BNC group and NBNC group was 70.2 and 69.7 years old respectively (p = 0.746) and general patients’ characteristic were listed in Table 1 and Additional file 1: Table S1. Three patients with BNC and one patient with NBNC had postoperative urinary tract infection and received oral antibiotics. One patient after ThuVep without BNC developed post-operative bleeding and blood clot retention and received further surgery.

**Fig. 1:**
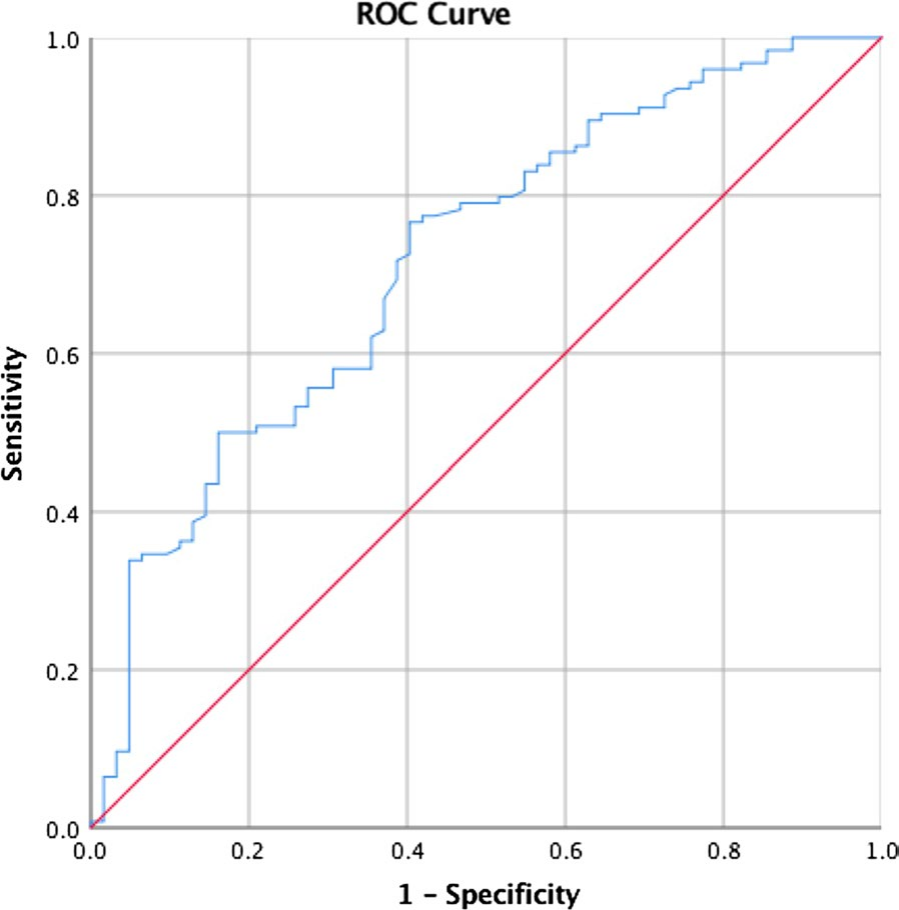
ROC curve for prostate volume to determine the cutoff value that predicts the occurrence of BNC after TURP or ThuP. Red line, reference line; blue line, prostate volume in cm^3^. Threshold: prostate volume: 42.9 cm^3^, AUC: 0.718, sensitivity: 0.766, specificity: 0.409

Univariate analysis showed that when compared with patients without BNC, those with BNC had significantly smaller prostates (43.0 ± 18.95 ml vs. 57.2 ± 19.84 ml, p < 0.001), lower resection weight (11.64 ± 11.75 g vs. 16.67 ± 12.84 g, p = 0.01), shorter operative times (43.0 ± 30.9 min vs. 57.2 ± 34.5 min, p = 0.001) and recatherization after surgery [yes/no, 7/55 vs. 2/122, p = 0.01, OR 5.6 (1.02–30.6)] (Table 2). Multivariate logistic analysis showed those with BNC had significant smaller prostate volume (OR 0.96 (0.94–0.99), p = 0.008) and high incidence of recatherization (OR 5.6 (1.02–30.6), p = 0.047) compared with those without BNC (Table 3). There were no significant differences in percentage of prostate resected, initial PSA, preoperative hemoglobin, postoperative hemoglobin, hemoglobin change, hospital stay, smoking history, current ESCL, maximal flow rate, post-voiding residual urine, postoperative urinary tract infection, and whether or not a Foley was inserted preoperatively. In subgroup analysis, those with BNC after TURP had significant smaller prostate volume and slower resection speed compared with NBNC group (Table 1). A ROC curve predicted that a prostate volume < 42.9 cm^3^ (AUC: 0.718, sensitivity: 0.766, specificity: 0.409) had a higher rate of BNC (Fig. 1).

**Table 2:** Univariate logistic regression analysis of incidence of BNC

	TURP	P value	ThuP	P value	Total	P value
	OR (95% interval)		OR (95% interval)		OR (95% interval)	
Prostate volume (ml)	0.95 (0.92–0.99)	0.007*	0.96 (0.94–0.99)	0.008*	0.96 (0.94–0.98)	< 0.001*
Maximal urinary flow (ml/s)	1.06 (1.001–1.12)	0.046*				
Prostate resection weight (g)	0.94 (0.89–0.99)	0.04*			1.02 (0.93–0.99)	0.01*
Surgical time (min)	0.98 (0.96–0.99)	0.01*	0.98 (0.97–0.99)	0.04*	0.98 (0.97–0.99)	0.02*
Resection speed (g/min)	0.002 (0.001–0.94)	0.048*	–	–	–	–
Cerebrovascular accidents (yes/no)					3.6 (1.3–9.8)	0.01*
Coronary artery disease (yes/no)			4 (1.2–12.8)	0.02*		
Chronic kidney disease (yes/no)			8.5 (2.1–34.9)	0.003*	3 (1.3–7.0)	0.007*
Number of comorbidities (≥ 2/ ≤ 1)			4.9 (1.7–13.4)	0.002*	3.2 (1.6–6.1)	0.001*
Recatherization after surgery (yes/no)					5.6 (1.6–38.6)	0.01*

* means  p < 0.05

**Table 3:** Multivariate logistic regression analysis of incidence of BNC

	TURP	P value	ThuP	P value	Total	P value
	OR (95% interval)		OR (95% interval)		OR (95% interval)	
Prostate volume (ml)	0.95 (0.92–0.99)	0.007*	0.96 (0.92–1)	0.07	0.96 (0.94–0.99)	0.008*
Maximal urinary flow (ml/s)	1.04 (0.98–1.1)	0.20				
Prostate resection weight (g)	1.04 (0.82–1.3)	0.74			1.02 (0.98–1.1)	0.67
Surgical time (min)	0.99 (0.96–1.03)	0.97	0.99 (0.97–1)	0.65	0.99 (0.98–1)	0.38
Resection speed (g/min)	0.027 (–)	0.78	–	–	–	–
Cerebrovascular accidents (yes/no)					1.6 (0.5–5.4)	0.42
Coronary artery disease (yes/no)			3 (0.6–14)	0.17		
Chronic kidney disease (yes/no)			7.3 (1.1–49.4)	0.04*	2.7 (0.9–7.9)	0.08
Number of comorbidities (≥ 2/ ≤ 1)			1.1 (0.2–5.2)	0.92	1.6 (0.7–3.8)	0.28
Recatherization after surgery (yes/no)					5.6 (1.02–30.6)	0.047*

* means  p < 0.05

Analysis of comorbidities showed that a greater proportion of BNC patients had history of cerebrovascular accidents [11/62 vs. 7/124, p = 0.009, OR 3.6 (1.3–9.8)], chronic kidney disease [14/62 vs. 11/124, p = 0.01, OR 3.0 (1.3–7.0)], and two or more comorbidities [29/62 vs. 27/124, p = 0.001, OR 3.2 (1.6–6.1)] (Tables 1 and 2). The presence of prostate cancer or other malignancies was not associated with risk of BNC. In ThuP subgroup analysis, a greater proportion of BNC patients had history of coronary artery disease [9/18 vs. 6/48, p = 0.02, OR 4.0 (1.2–12.8)], chronic kidney disease [9/18 vs. 3/51, p = 0.001, OR 8.5 (2.1–34.9)], and two or more comorbidities [15/12 vs. 11/43, p = 0.001, OR 4.9 (1.6–13.4)] (Tables 1 and 2). However, multivariate logistic regression analysis showed no significant difference in comorbidities. In ThuP subgroup analysis, a greater portion of BNC patients had history of chronic kidney disease [p = 0.04, OR 7.3 (1.1–49.4)] (Table 3).

In order to evaluate whether different surgical techniques were associated with the incidence of BNC, a 1:1 propensity score matching model according to age, prostate volume and comorbidity was built and propensity score was set at 0.01 (Table 4). 49 matched pairs were evaluated and there was no significant difference in all parameters (p > 0.05). Logistic regression analysis showed there was no significant difference in incidence of BNC between resection and enucleation group (p = 0.5).

**Table 4:** Propensity score match-controlled (1:1) cohort by age, prostate volume and comorbidity

	All patients			Cohort		
	Resection group (N = 135)	Enucleation group (N = 51)	P value	Resection group (N = 49)	Enucleation group (N = 49)	P value
Age (years)	68.8 ± 10.3	72.9 ± 7.4	0.049*	71.6 ± 8.1	72.5 ± 7.2	0.44
Prostate volume (ml)	51.02 ± 20.72	56.37 ± 19.90	0.72	57.40 ± 23.59	54.90 ± 18.87	0.42
PSA (ng/ml)	8.77 ± 15.22	8.47 ± 13.51	0.57	8.08 ± 7.90	7.88 ± 13.4	0.88
Maximal urinary flow (ml/sec)	9.44 ± 7.33	8.47 ± 13.51	0.58	8.46 ± 5.83	8.10 ± 6.41	0.79
Post-voiding residual urine (ml)	129.28 ± 171.40	119.94 ± 167.55	0.45	124.20 ± 156.88	119.88 ± 170.97	0.20
Foley insertion before TURP or ThuP (yes/no)	26/109	10/41	0.96	9/40	9/40	1.0
Pre-operation hemoglobin (ng/ml)	14.06 ± 1.43	13.99 ± 1.11	0.12	14.06 ± 1.60	14.02 ± 1.11	0.49
Post-operation Hemoglobin (ng/ml)	13.41 ± 1.47	13.29 ± 1.08	0.01*	13.28 ± 1.68	13.31 ± 1.07	0.86
Hemoglobin change (ng/ml)	0.66 ± 0.64	0.68 ± 0.71	0.82	0.77 ± 0.74	0.69 ± 0.72	0.13
Prostate resection weight (g)	14.64 ± 11.69	16.10 ± 15.10	0.045*	17.81 ± 13.65	15.33 ± 14.33	0.14
Percentage of prostate resected (%)	27.77 ± 14.93	26.65 ± 20.57	0.015*	30.10 ± 14.75	26.21 ± 20.45	0.71
Surgical time (min)	95.71 ± 32.76	111.69 ± 35.43	0.49	102.67 ± 29.92	110.33 ± 35.47	0.75
Hospital stay (days)	3.13 ± 0.50	3.14 ± 0.49	0.31	3.06 ± 0.48	3.14 ± 0.50	0.80
Smoking (yes/no)	48/87	10/41	0.036*	15/34	10/39	0.33
Hypertension (yes/no)	65/70	26/25	0.73	25/24	24/25	1.0
Diabetes mellitus (yes/no)	25/110	7/44	0.44	9/40	7/42	0.79
Cerebrovascular accidents (yes/no)	12/123	6/45	0.55	5/44	5/44	1.0
Coronary artery disease (yes/no)	22/113	9/42	0.83	10/39	8/41	0.82
Chronic kidney disease (yes/no)	16/119	9/42	0.30	8/41	8/41	1.0
Number of comorbidities (≥ 2/ ≤ 1)	38/97	18/33	0.34	15/34	16/33	1.0
Prostate cancer (yes/no)	15/120	3/48	0.28	5/44	3/46	0.73
Other malignancy (yes/no)	9/126	1/50	0.20	3/46	1/48	0.63
Concurrent ESCL (yes/no)	9/126	1/50	0.20	3/46	1/48	0.63
Recatherization after surgery (yes/no)	4/131	5/46	0.052	1/48	5/44	0.22
Postopertive urinary tract infection (yes/no)	3/132	1/50	0.91	1/48	1/48	1
BNC (yes/no)	45/90	17/34	1	13/36	16/33	0.69

* means  p < 0.05

In our study, six patients after TURP, three patients after ThuVap and two patients after ThuVep or ThuEp had recurrent BNC and received bladder neck incision two times. One patient after TURP and one patient after ThuVap had recurrent BNC and received bladder neck incision three times.

## Discussion

BPH with lower urinary tract symptoms is a common problem in older males. TURP is still considered the gold standard surgical treatment, and ThuP and Holmium laser enucleation of prostate (Holep) have recently been incorporated into national and international guidelines due to their noninferior efficacy and safety [8]. BNC is a common postoperative complication that typically occurs early within the first 2 years post-operation [9]. Our study showed average time to a diagnosis of BNC was 21.3 months. A meta-analysis reported that the incidence of BNC was 2% [10]. Another study showed that TURP resulted in BNC rates between 1% and 12.3% [2]. The incidence of BNC reported after ThuVeP ranges from 0 to 2.4% [11, 12]. The incidence of BNC after Holep is between 1.3 and 2.1% [13–15]. Our study results were comparable with these previous studies as the incidence of BNC was 1.5% after TURP and 1.6% after ThuP (2.2% after ThuVap (10/458), and 1.4% (17/1198) after ThuVep or ThuEp).

BNC after prostate surgery is a well-known complication but its underlying cause is not well understood. Many risk factors, including small prostate volume, higher International Prostate Symptom Score storage scores, preoperative uncontrolled infection, unsuitable resectoscope, large resection loop, extensive resection of the bladder neck, long surgical time and recatherization after surgery have been shown to be associated with the risk of BNC [6–8]. The most reported risk factor is small prostate volume with over-resection of the bladder neck. Over-resection of the bladder neck can lead to fibrosis or scarring of the bladder neck, which in turn leads to BNC. Chiu et al.reported that the incidence of BNC increased to 16% in patients with a prostate volume < 20 g [16]. Our study showed that a prostate volume < 42.9 cm^3^ had a higher rate of BNC. Low volume of prostate resected and short surgical time were positively correlated with small prostate volume during TURP. Resection speed is a better parameter as it adjusts for resected prostate weight and surgical time. We hypothesized slow resection speed was correlated with unfavorable surgical processes, such as hemorrhage, poor endoscopic vision, prolonged operative time and perforation of the prostatic fossa or bladder neck. In these cases, more meticulate and extensive hemostasis using a bipolar resection loop will likely be performed and this can lead to a higher chance of thermal injury of the bladder neck, which increases the risk of bladder neck scarring. However, our study showed a lower resection speed in BNC group compared to NBNC group in TURP (OR 0.002 (0.001–0.94), p = 0.048) but no significant difference in pre- and post-operative hemoglobin and hemoglobin change (Tables 1 and 2).

A randomized controlled study reported that ThuEp significantly reduced the risk of BNC compared with ThuVap [17]. Another study reported that the incidence of BNC after plasmakinetic enucleation of prostate was only 0.9% [18]. Qian et al. suggested that enucleation of the prostate can keep the bladder neck intact and prevent injury caused by laser energy, which in turn reduces scar formation and decreases the development of BNC [17]. In our study, the propensity score matching model showed no difference in the incidence of BNC between resection and enucleation groups, which could be related to the use of bipolar devices for final hemostasis in both TURP and ThuP.

We hypothesized that preoperative comorbidities and smoking history are potential risk factors for BNC. Microvascular disease may be associated with BNC due to poor healing and local ischemia [19]. Development of microvascular disease, such as diabetes mellitus, coronary artery disease, cerebrovascular disease, hypertension, chronic kidney disease and smoking history could theoretically alter the microvascular blood supply in the bladder neck, and accompanied by local ischemia and the wounds caused by TURP or ThuP, could lead to scar formation. In the current study, chronic kidney disease (p = 0.007) and cerebrovascular accidents (p = 0.01) were associated with increased BNC risk. In addition, the presence of ≥ 2 comorbidities was a significant risk factor (p = 0.001) and 3.2 for BNC, which could indicate that there is a correlation between microvascular disease and BNC (Table 2).

Foley insertion before surgery, postoperative urinary tract infection and repeated catherization after surgery could be possible unfavorable factors related to inflammation or trauma in the urinary tract, which can cause scar formation in the urethra and bladder neck. Tao et al. reported that post-operative urinary tract infection and recatherization were correlated to urethra stricture and not correlated to BNC [7]. Grechenkov et al. reported that repeated postoperative drainage of bladder [6]. Our study reported that recatherization was related to BNC (OR 5.6 (1.02–30.6), p < 0.047) while Foley insertion before surgery and postoperative urinary tract infection were not correlated to BNC.

A prophylactic incision of the bladder neck using a bipolar loop or laser at the end of surgery, may reduce the incidence of BNC [7]. Incision of the bladder neck by laser is preferred [20]. Dysuria is the primary symptom of BNC, and a positive diagnosis is confirmed by cystoscopy. Urethral dilation is a potential management tool for BNC but repeat urethral dilation due to recurrent BNC was observed in 90% of patients in the first two years [21]. BNC is managed by bladder neck incision, which has a 72% success rate [22]. In our study, 62 patients with BNC received bladder neck incision via bipolar instruments, which has a 79% (49/62) success rate. 13 patients had recurrent bladder neck contracture and received bladder neck incision. Refractory BNC presents as recurrent dysuria in a short time and may require repeat bladder neck incision. Another technique combines bladder neck incision with a transurethral irrigation agent or transurethral injection of a cytotoxic agent. Eltahawy et al. reported an 83% success rate for the combination of bladder neck incision via Holmium laser and irrigation with triamcinolone, while Redshaw et al. showed a 75% success rate for bladder neck incision using a cold-knife and transurethral injection of mitomycin C [23, 24].

This study had several limitations. First, we did not use the International Prostate Symptom Score as a parameter in the study due to incomplete medical records. Preoperative Foley status, maximal flow rates and post-voiding residual urine were not significant different between the BNC and NBNC patients (p = 0.43, p = 0.05 and p = 0.39). Second, it was a single center, retrospective study, the number of patients was small and selection bias would be existent. Third, some patients were lost to follow-up years after TURP or ThuP, which could lead to an underestimation of the BNC incidence rates. There was the same size of resectoscope, resection loop and energy settings, and no previous endourological interventions, all of which minimized bias in the present study.

Our study demonstrated that low prostate volume, recatherization and the presence of ≥ 2 comorbidities were positively correlated with the development of BNC after TURP or ThuP and the incidence was the same in resection and enucleation groups. Low resection speed was positively correlated with the development of BNC after TURP. A small prostate volume less than < 42.9 cm^3^ had a higher rate of BNC. However, larger studies are needed to verify these results. Our study may serve as reference for clinical urologists and our results can be used during the explanation of BNC risks before surgery.

## Supplementary Information

**Additional file 1. Table S1**: Patients’ characteristics in ThuP subgroups

## Data Availability

Records and data pertaining to this study are in the patient’s secure medical records in Mackay Memorial Hospital and are available from the corresponding author on reasonable request. All methods were performed in accordance with the relevant guidelines and regulations and approved by the Institutional Review Board of Mackay Memorial Hospital.

## Abbreviations

PSA: Prostate specific antigen
BPH: Benign prostate hyperplasia
TURP: Bipolar transurethral resection of prostate
ThuVap: Thulium vaporesection
ThuVep: Thulium vapoenucleation of the prostate
ThuEp: Thulium enucleation of the prostate
ThuP: Transurethral surgery of the prostate with Thulium laser
BNC: Bladder neck contracture
ESCL: Concomitant endoscopic cystolithotripsy
Holep: Holmium laser enucleation of prostate
