# Supplementary material for: Analysis of risk factors of bladder neck contracture following transurethral surgery of prostate
Source: BMC Urol. 2021 Apr 11;21:59. doi: 10.1186/s12894-021-00831-6 (PMC8037916; doi:10.1186/s12894-021-00831-6)
Supplement: Supplementary file 1 — Additional file 1. Table S1: Patients’ characteristics in ThuP subgroups [file 12894_2021_831_MOESM1_ESM.docx]

Supplementary Table 1: Patients’ characteristics in ThuP subgroups

|  | ThuVap | | P value | ThuVep+ThuEp | | P value |
| --- | --- | --- | --- | --- | --- | --- |
|  | BNC  (N=10) | NBNC  (N=20) |  | BNC  (N=17) | NBNC  (N=34) |  |
| Age (years) | 67.45±7.16 | 70.4±8.47 | 0.36 | 74.6±7.27 | 72.06±7.48 | 0.25 |
| Prostate volume (ml) | 39.00±14.43 | 61.44±22.83 | 0.009* | 50.72±17.03 | 59.20±20.86 | 0.15 |
| PSA (ng/ml) | 13.35±27.87 | 9.52±16.24 | 0.64 | 10.82±20.34 | 7.29±8.48 | 0.38 |
| Maximal urinary flow (ml/sec) | 10.71±6.54 | 11.15±4.98 | 0.84 | 8.68±7.37 | 7.41±5.97 | 0.51 |
| Post-voiding residual urine (ml) | 43.92±45.83 | 84.86±124.75 | 0.33 | 149.27±172.62 | 105.28±165.60 | 0.38 |
| Foley insertion before TURP or ThuP(yes/no) | 1/9 | 3/17 | 0.70 | 4/13 | 6/28 | 0.62 |
| Preoperative Hemoglobin(gm/dl) | 13.63±1.49 | 13.94±1.37 | 0.57 | 13.64±0.59 | 14.12±1.27 | 0.11 |
| Postoperative Hemoglobin(gm/dl) | 13.3±1.28 | 12.99±1.38 | 0.54 | 12.81±0.55 | 13.52±1.21 | 0.02* |
| Hemoglobin change(gm/dl) | 0.32±0.38 | 0.96±0.64 | 0.008* | 0.75±0.50 | 0.64±0.80 | 0.63 |
| Prostate resection weight (g) | 14.63±11.69 | 16.10±15.10 | 0.49 | 16.24±17.58 | 16.03±13.99 | 0.96 |
| Percentage of prostate resected (%) | 27.77±14.93 | 26.65±20.57 | 0.68 | 29.77±27.19 | 25.09±16.60 | 0.45 |
| Surgical time (minutes) | 95.71±32.76 | 111.69±35.43 | 0.004* | 104.24±37.10 | 115.41±34.60 | 0.29 |
| Hospital stay(days) | 3.13±0.50 | 3.14±0.49 | 0.96 | 3.06±0.24 | 3.18±0.58 | 0.43 |
| Smoking (yes/no) | 1/9 | 5/15 | 0.33 | 6/11 | 4/30 | 0.046* |
| Hypertension (yes/no) | 4/6 | 11/9 | 0.44 | 13/4 | 13/21 | 0.01* |
| Diabetes mellitus (yes/no) | 3/7 | 5/15 | 0.77 | 4/13 | 3/31 | 0.15 |
| Cerebrovascular accidents (yes/no) | -/10 | -/20 | - | 4/13 | 2/32 | 0.07 |
| Coronary artery disease (yes/no) | 3/7 | 3/17 | 0.33 | 6/11 | 3/31 | 0.02* |
| Chronic kidney disease(yes/no) | 1/9 | 2/18 | 1 | 8/9 | 1/33 | <0.001* |
| Number of Comorbidities (≥2/≤1) | 3/7 | 5/15 | 0.77 | 12/5 | 6/28 | <0.001* |
| Prostate cancer (yes/no) | 1/9 | 2/18 | 1 | 1/16 | 2/32 | 1 |
| Other malignancy (yes/no) | 1/9 | 0/20 | - | 0/17 | 1/33 | - |
| Concurrent ESCL(yes/no) | 0/10 | 3/17 | - | 0/17 | 1/33 | - |
| Recatherization after surgery(yes/no) | 0/10 | 0/20 | - | 3/14 | 2/32 | 0.18 |
| Post-opertion urinary tract infection(yes/no) | 1/9 | 0/20 | - | 1/16 | 0/34 | - |
